# Chrysotile effects on human lung cell carcinoma in culture: 3-D reconstruction and DNA quantification by image analysis

**DOI:** 10.1186/1471-2407-8-181

**Published:** 2008-06-27

**Authors:** Beatriz A Cortez, Glaucia M Machado-Santelli

**Affiliations:** 1 Dept. of Cell and Developmental Biology, Institute of Biomedical Sciences, University of Sao Paulo, Av. Lineu Prestes 1524, 05508-000, Sao Paulo, Brazil; 2 Dept. of Genetic and Evolutive Biology, Biosciences Institute, University of Sao Paulo, Sao Paulo, Brazil

## Abstract

**Background:**

Chrysotile is considered less harmful to human health than other types of asbestos fibers. Its clearance from the lung is faster and, in comparison to amphibole forms of asbestos, chrysotile asbestos fail to accumulate in the lung tissue due to a mechanism involving fibers fragmentation in short pieces. Short exposure to chrysotile has not been associated with any histopathological alteration of lung tissue.

**Methods:**

The present work focuses on the association of small chrysotile fibers with interphasic and mitotic human lung cancer cells in culture, using for analyses confocal laser scanning microscopy and 3D reconstructions. The main goal was to perform the analysis of abnormalities in mitosis of fibers-containing cells as well as to quantify nuclear DNA content of treated cells during their recovery in fiber-free culture medium.

**Results:**

HK2 cells treated with chrysotile for 48 h and recovered in additional periods of 24, 48 and 72 h in normal medium showed increased frequency of multinucleated and apoptotic cells. DNA ploidy of the cells submitted to the same chrysotile treatment schedules showed enhanced aneuploidy values. The results were consistent with the high frequency of multipolar spindles observed and with the presence of fibers in the intercellular bridge during cytokinesis.

**Conclusion:**

The present data show that 48 h chrysotile exposure can cause centrosome amplification, apoptosis and aneuploid cell formation even when long periods of recovery were provided. Internalized fibers seem to interact with the chromatin during mitosis, and they could also interfere in cytokinesis, leading to cytokinesis failure which forms aneuploid or multinucleated cells with centrosome amplification.

## Background

Asbestos, the general name given to six different fibrous silicate minerals, are divided into two groups of fibers: amphiboles and serpentines. Amphibole fibers had been mostly used by the market in the past, until being associated with several serious health diseases. They are causally related to the development of asbestosis, bronchial cancer, malignant mesothelioma of pleura and peritoneum, and, to a more limited extent, to various gastrointestinal, oropharyngeal and laryngeal cancers [1,2]. Nowadays amphibole fibers cannot be commercialized in many countries and have been replaced by serpentine fibers, mainly by chrysotile, which accounts for more than 95% of asbestos found in United States buildings.

Chrysotile is characterized by curves and silken fibers, small transversal section (180 a 300Å) and tubular structure, and it is considered less harmful to human health. Its clearance from the lung is faster than it is with amphibole fibers, for chrysotile asbestos fails to accumulate in the lung tissue due to a mechanism involving fibers fragmentation in short pieces. Short exposure to chrysotile has not been associated with any histopathological alteration of lung tissue, in contrast to amphibole exposure, which is related to inflammatory response, granuloma and mild interstitial fibrosis [3,4]. Since genotoxicity is a general prerequisite for the development of malignancy, a great amount of data, collected in several end-point tests, has shown that the exposure to asbestos fibers results in chromosomal aberrations and mutations. Together with the solid epidemiological evidences, these data confirm the asbestos as a carcinogenic agent. However, the mechanism by which asbestos produce malignancy is unclear at the present moment [5]. According to Walker et al. (1992) [6] this mechanism would involve direct and indirect effects: the physical interaction of fibers with target cells or the free radicals generation from the fiber surface acting directly on DNA and indirectly on inflammatory reactions.

Chrysotile toxicity and mutagenicity have also been evaluated. It has been shown that chrysotile fibers induce chromosome aberrations in human lymphocytes from whole blood cultures, peritoneal fluid cells and bone marrow cells of mice [7]. The fibers were able to induce structural and numerical chromosomal aberrations in human amniotic fluid cells, increasing the number of hyperdiploid cells in treated cell population [8]. Aberrant mitosis and multi-polar spindles observed in asbestos treated cells could lead to the incorrect chromosome segregation and result in aneuploid cells [9,10]. The loss or gain of even one single chromosome, or part of it, can introduce multiple mutations required for the acquisition of malignant phenotypes [11]. Aneuploidy has also been associated with tumor progression since the majority of solid human tumors are non-diploid [12-14].

Chromosome instability can also be caused by loss of mitotic checkpoint functions, imperfections in kinetochore functions and centrosome amplification. The centrosome amplification is classically associated with aneuploidy. Correct chromosome segregation depends on the presence of two centrosomes and bipolar spindles. In this context, the centrosome amplification leads to mitotic and cytokinesis errors by the formation of multi-polar spindles [15]. The mechanisms involved in centrosome amplification and the relationship between the cell and the centrosome cycle regulation seems to be strongly connected, since some proteins are involved in both cycles, like cdk2 and cyclin E, which promote the entry in S and the centrosome duplication [16-18].

The present work focuses on the association of small chrysotile fibers with interphasic and mitotic human lung cancer cells in culture, using for analyses confocal laser scanning microscopy and 3D reconstructions. The main goal was to perform the analysis of abnormalities in mitosis of fibers-containing cells as well as to quantify nuclear DNA content of treated cells during their recovery in fiber-free culture medium.

## Methods

### Cell culture

The HK2 cell line established from human non-small cell lung carcinoma [19] were cultured in Dulbecco's Modified Eagle's Minimum Essential Medium (Sigma), supplemented with 10% fetal bovine serum, in a humidified atmosphere with 5% CO_2 _at 37°C.

### Treatment with chrysotile

Chrysotile 5R (Quebec Standard) obtained from SAMA Mineração de Amianto Ltda (Minaçu, GO, Brazil) were kindly provided by Dr. Flavia M. Cassiola. The fibers were washed with tap water and activated by sonication at controlled pH (7.4) as described elsewhere [20]. For treatment, cells were enzymatically removed from the flasks and plated in 35 mm diameter dishes (2.10^5 ^cells/dish) containing a glass coverslip. After 24 h in culture, the medium was changed to 2 mL of fresh medium with chrysotile fibers at an approximated final concentration of 0.25 mg/mL. The fibers remained in contact with the cells for a period of 48 h, after which the medium was changed. After additional periods of 24, 48 or 72 hours, cells were washed with PBSA three times and fixed with formaldehyde 3.7% for 30 min. During all the treatment the medium culture used was supplemented with 10% fetal bovine serum.

### Immunoflourescence, Laser Scanning Microscopy and 3D reconstruction

After chrysotile treatment and recovery, the culture medium was removed and the cells in suspension were recovered by centrifugation. The dettached and adherent cells were submitted to immunofluorescence independently. Adherent cells were fixed with formaldehyde 3.7% for 30 min, washed three times with PBSA and permeabilized with Triton X-100 0.5% for 10 min. Then, the cells were treated with RNAase for 30 min and incubated with the primary antibody (anti-γ-tubulin, Sigma) diluted 1:800, or anti-β-tubulin (Sigma, diluted 1:200) overnight. After that, the cells were washed three times with PBSA and incubated with 2nd antibody (anti-mouse FITC or Cy5, diluted 1:200) for 2 h. The nuclei were stained with propidium iodide and actin filaments with FICT-phalloidin for 20 min. Detached cells were fixed with formaldehyde 3.7% for 30 min, washed with PBSA, and spun down on poly-lysine coated glass slides with the use of a cytological centrifuge (3.10^4^cell/slide, 1000 rpm for 2 min). After treatment with Triton X-100 0.5% for 10 min and RNAase for 30 min, the nuclei were stained with propidium iodide.

The cell preparations were analyzed by confocal laser scanning microscopy (Zeiss LSM 510) with the use of a 40X objective. Optical sections of 0.5–1 μm were used for 3D reconstructions made by the software Imaris 3.1.3 (Bitplane AG), in a Silicon Graphics station. Imaging of chrysotile fiber was performed in a confocal configuration to detect its autofluorescence (excitation with Argon laser at 488 nm, HFT 488 filter; for details see Additional file 1).

### DNA Ploidy

The nuclear DNA content was quantified by image analysis with the software CIRES (Cell Image Retrieval and Evaluation System-Kontron Eletronik) installed in Axioskop microscope (Zeiss). For the analysis, the nuclei were stained by Feulgen's reaction [12]. The DNA from nuclei of mononucleated, binucleated and multinucleated cells was quantified independently both in control and in chrysotile treated cells. Three independent experiments were done, and more than 500 nuclei for each treatment and control cells were analyzed, with the use of a 40X objective.

### Statistical Analysis

The results were analyzed by χ^2 ^test and P < 0.01 was considered significant. The differences of frequency of apoptotic, mononucleated, binucleated and multinucleated cells were tested, between the control situation and after the chrysotile treatment, considering the three periods used of recovery in normal medium.

## Results

### Multinucleation and apoptosis induction after chrysotile treatment

Since this study focuses the interaction of chrysotile fibers with cells in culture, the most important aspect was to observe a large number of cells with internalized fibers independently of the fiber concentration in culture medium. The final fiber concentration in the culture medium is difficult to be determined, once added to fetal bovine serum supplemented culture medium some fibers float. Those fibers do not interact with the cell, and they were removed from the culture with the medium changing after 48 h treatment. However, it was observed that cell treatment with 0.25 mg/mL of chrysotile resulted in high frequency of cells interacting with fibers of 0.3 to 30 μm length and it represents a situation of high fibers exposure.

**Figure 1:**
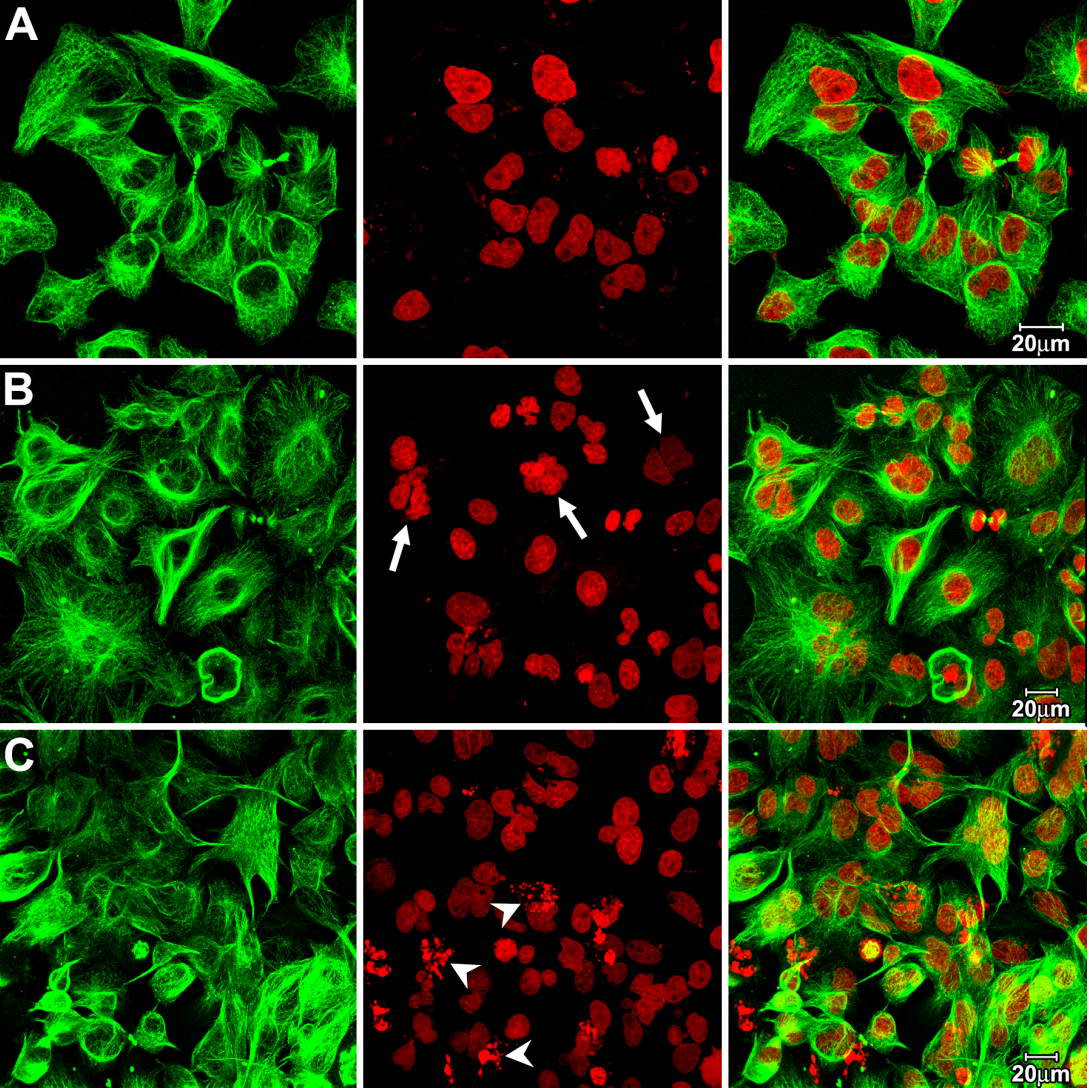
**HK2 cells in control situation and after 48 h chrysotile exposure**. Confocal images of HK2 cells, showing microtubules (green) and DNA stained by PI (red). **A) **Control HK2 cells were predominantly mononucleated and apoptosis were rarely observed. **B and C) **HK2 cells after treatment with chrysotile for 48 h and additional 24 h in fiber-free medium show high frequency of multinucleated cells (arrows) and apoptosis (arrows tips). The pattern of microtubules in interphasic treated cells is similar to control cells, however in multinucleated cells a reorganization of cytoskeleton in a radiated distribution was observed.

HK2 cells treated with chrysotile for 48 h and recovered for additional 24 h in normal culture medium showed increased frequency of morphological nuclear alterations. Chrysotile treated cells acquired some features rarely observed in control cells (Figure 1A), such as the presence of multinucleated and apoptotic cells (Figure 1B and 1C). In general, the patterns of microtubules and microfilaments distribution in interphasic cells were similar to those of control cells (Figure 1B and 1C). However, the multinucleated cells in treated preparations presented microtubules with a radiated distribution, similar to giant tumor cells (Figure 1B).

Control cell population was formed basically by mononucleated cells (83.4%) and the presence of apoptotic cells was rarely observed. The frequency of cells with two or more nuclei increased from about 15% in control cells to 45% in chrysotile-treated cells, reaching approximately 65% after longer recovery time (*P *< 0.01, Table 1). Apoptotic cells, identified by the presence of fragmented chromatin, represented 0.26% of control cells. During the treatment lot of cells detached from the substrate and 34% of these cells showed apoptotic morphology after 24 h of recovery, in addition to 6% of apoptotic cells in monolayer (*P *< 0.01, Table 1).

**Table 1:** Analyses of DNA content in control and chrysotile treated HK2 cells, and frequency of apoptosis and multinucleation.

		Frequency (%)	DNA index (C)	Max. DNA (C)	DNA index > 5C (%)	Apoptosis (%)
CONTROL	mono	83.40 (563/675)	1.3	7.0	0.5	-
	bi	10.97 (74/675)	1.1	4.7	0	-
	multi	5.63 (38/675)	0.9	4.3	0	-
			1.2	7.0	0.2	0.26 (2/771)
48 h chrysotile + 24 h recovery	mono	54.07 (259/479)	1.6	8.9	3.5	-
	bi	24.64 (118/479)	1.4	8.9	2.1	-
	multi	21.29 (102/479)	1.2	8.5	3.1	-
			1.4	8.9	2.9	4.64 (30/646)
48 h chrysotile +48 h recovery	mono	34.65 (175/505)	1.7	10.2	6.0	-
	bi	29.11 (147/505)	1.6	9.8	7.5	-
	multi	36.24 (183/505)	1.4	14.5	8.8	-
			1.6	14.5	7.4	6.36 (32/503)
48 h chrysotile +72 h recovery	mono	35.57 (180/506)	1.8	17.7	10.8	-
	bi	22.73 (115/506)	1.6	14.6	10.0	-
	multi	41.70 (211/506)	1.5	14.1	10.0	-
			1.6	17.7	10.2	2.58 (13/504)

After chrysotile treatment the cell culture acquired some features, such as increasing of apoptotic, binucleated and multinucleated cells. The software used to quantify the DNA content made possible to verify the percentage of nuclei with DNA content higher than 5C, and to observe the minimum and maximum values of DNA content in all classes analyzed.

### Nuclear DNA quantification

Nuclear DNA contents of mononucleated, binucleated and multinucleated control and treated cells were independently quantified by image analysis. In this series of experiments the treatment consisted in the exposure to chrysotile fibers for 48 h followed by three period of recovery in normal culture medium: 24 h, 48 h and 72 h.

**Figure 2:**
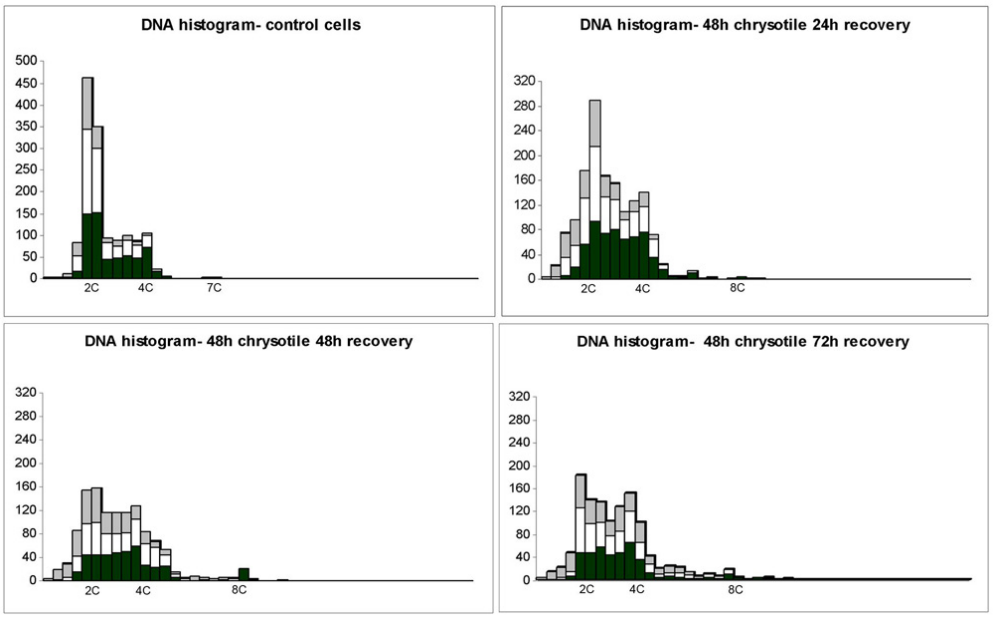
**DNA Histograms of control and chrysotile treated cells**. The nuclear DNA content from control and chrysotile treated cells was quantified independently by image analysis with the software CIRES. In DNA histograms (N vs. C plot) the nuclei of mononucleated cells are represented in black, the nuclei of binucleated cells are in white and the nuclei of multinucleated cells are in gray, and after 48 h chrysotile treatment were used three different times of recovery in free-fiber medium (24 h, 48 h and 72 h).

The distribution of the nuclear DNA content of control cells showed a main peak in 2C region (DNA index = 1), and the values were distributed mainly between 2C and 4C, for mononucleated, bi or multinucleated cells. After chrysotile treatment and 24 h recovery the peak in 2C is not so evident, and most of mononucleated cells presented DNA content > 2C till 4C. Nuclei of bi and multinucleated cells distributions were similar, with a slightly higher frequency of 2C cells than in mononucleated cells. In 48 h and 72 h recovery the frequency 2C cells decreased, and the number of cells with DNA values > 5C increased progressively from 0.2 to 10% (Figure 2). It is interesting to observe that the > 5C nuclei frequency seems to be dose-dependent. When cells were treated with 0.125 mg/mL and 0.0625 mg/mL chrysotile during 48 h plus 48 h recovery the > 5C nucleus frequencies were 4.8% and 3.8%, respectively (data not shown). The highest DNA values observed in each cell population also increased progressively in the different time of recovery from 7C to 17C, corresponding to very abnormal nucleus. These data are summarized in Table 1.

### Cell-fiber interaction

The cell-fiber interaction was analyzed by confocal microscopy and 3D reconstruction of HK2 cells exposed to chrysotile fibers for 48 h and maintained 24 h in normal medium. Cells in different phases of cell cycle presented both long chrysotile fibers and small fragment of fibers internalized.

**Figure 3:**
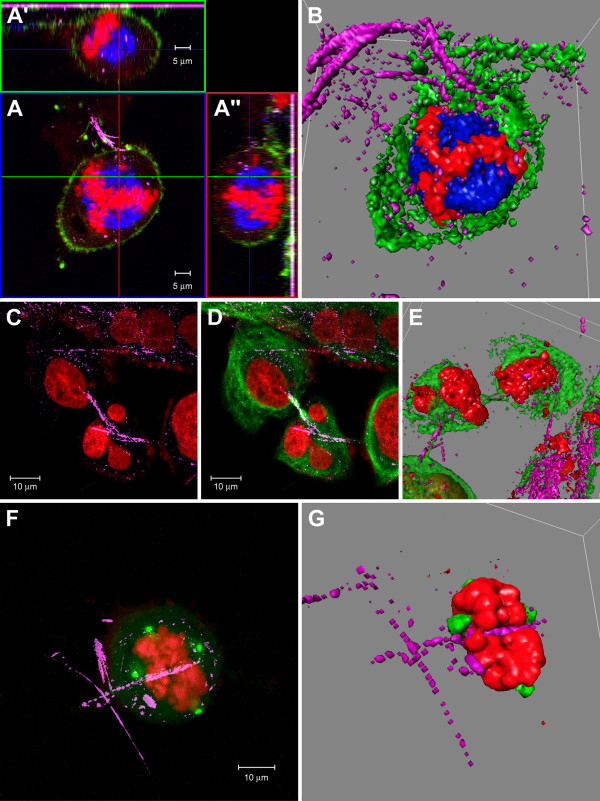
**Chrysotile treated cells during cell cycle**. Laser scanning **c**onfocal microscope images of HK2 cells treated with chrysotile for 48 h and recovered for 24 h in fiber-free culture medium: **A) **metaphase cell with multipolar spindle (microtubules in blue, DNA in red, actin filaments in green), presenting small fragments of chrysotile (pink) inside; **A') **and **A") **orthogonal projections showing the intracellular localization of chrysotyle fibers; **B) **3D reconstructions of optical sections of the same cell; small chrysotile fibers are also seen outside the cell (small pink dots). Chrysotyle treated HK2 cell in late telophase with a chrysotyle fiber between the two daughter cells: **C) **Image of nuclei in red and crysotyle fibers in pink, **D) **merge with microtubules (green) image; the microtubules seem to be normally organized, forming the midbody in cytokinesis region; **E) **the 3D reconstruction of their optical sections evidence the cell- fiber interaction, with the fiber inside the cell and between the daughter nuclei (red). **G **and **F) **Confocal image showing four centrosomes evidenced by anti-gamma tubulin (green) and condensed mitotic chromosomes (red). Chrysotile fibers (pink) are observed inside the cell in confocal image. The 3D reconstruction (**G**) evidence the fiber involved by chromatin.

Multi-polar spindles were observed in more than 50% of metaphasic/anaphasic cells in chrysotile treated cells. In contrast, in control cells the frequency of multi-polar metaphases was around 5%. The images of multipolar mitotic cells show small fibers internalized, observed in 3D reconstructions, and some of the small fragments were interacting with the chromatin (Figure 3A–B).

Fibers inside the cells in anaphase and late telophase were frequently located between the two daughter nuclei, and could also be associated with chromatin. Some of the cells in late telophase exhibited fibers in the intercellular bridge, between the daughter cells. In these cases the microtubules seemed to be organized, forming the midbody despite the presence of the fiber (Figure 3C–E). Aberrant telophase and cytokinesis, like the divisions resulting in three daughter cells, were also observed, and fibers in these cells were visualized mainly among the daughter cells. It is relevant to notice that cells in final phases of mitotic division were observed in greater frequency in control cells rather than in chrysotile treated cells.

With the presence of aneuploid cells and cells with multipolar spindles, the observation of centrosomes was important to the analyses of cell cycle disruptions. The immunofluorescence with anti-γ-tubulin antibody permitted the analyses of the centrosome number in mitotic cells, and metaphase and anaphase cells with more than two centrosomes were observed in frequency similar to cells with multipolar spindles. However, cells in metaphase with more than 4 centrosomes and also with 6 centrosomes per cell were observed. Small chrysotile fragments and long fibers interacting with the chromatin were also observed in these cells (Figure 3F–G).

**Figure 4:**
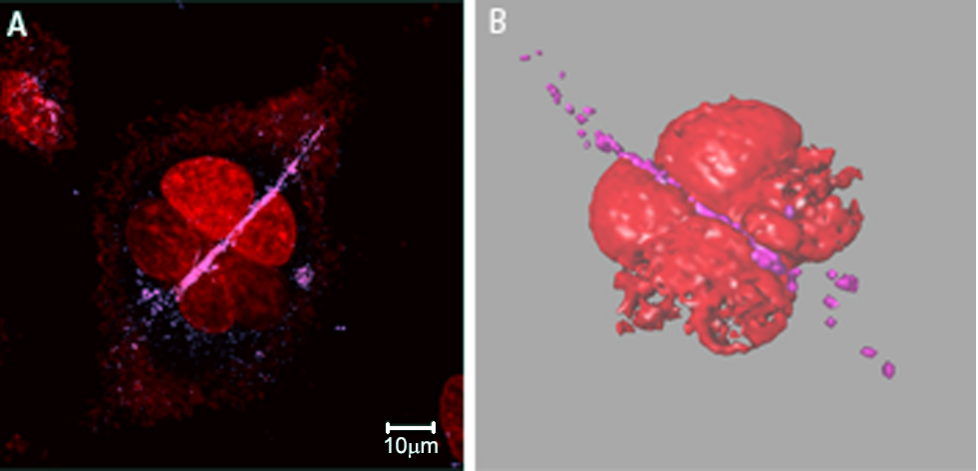
**Interphase multinucleate HK2 cell with a phagocytized chrysotile fiber**. **A) **Confocal image of a HK2 cell after 48 h chrysotile exposure showing an internalized fiber (pink) interacting with the nuclei (red, stained with PI). The image stacks were used for 3D reconstruction **(B)**, evidencing the spatial interaction between cell and fiber, where the fiber seems to segment the nucleus.

Chrysotile fibers in interphasic cells were normally located in perinuclear region, and also in the middle of the nuclei in multinucleated cells (Additional file 2). However, some fibers seem to be interacting with the nucleus (Figure 4A–B). In interphasic cells the number of centrosomes varied from one to four.

## Discussion

Chrysotile has been considered a quite safe type of fiber due mainly of the lack of evidence of its association with health problems and due to biopersistence data, indicating its fast elimination from lung tissues. However, the present data show that at least small fibers remain associated with cells after the chrysotile-containing medium is changed to normal culture medium. An interesting system to study the effect of fibers in a clearance-like period of time would be the "in vitro" treatment followed by recovery periods. Additionally, 3D reconstruction of confocal images, detecting fibers by their own auto-fluorescence, represented an important approach in the study of cell-fibers interaction, and evidenced fibers inside the cytoplasm and closely associated with chromatin. The presence of fibers in the intercellular bridge during cytokinesis causes a delay in the finalization of mitosis and could lead to abnormalities in daughter cells. The presence of fibers inside the mitotic cells was previously showed by different techniques in other cell types [21,22]. Together, this kind of data support the hypothesis proposed to fibers carcinogenicity mechanism based on mitosis physical interference of fibers resulting in chromosomal instability. Other proposed mechanisms for fibers carcinogenicity were: chronic inflammatory reaction induced by fiber contact with enhanced cytokines and growth factors production; catalysis of reactive radical species (such as ROS) resulting in direct or indirect DNA mutations [23,24].

In this study were used human lung cancer cells, which have mutations required for acquisition of malignant phenotypes. The mutations might interfere in the mitotic checkpoints functions, but probably do not interfere in the cell-fiber interaction, like the fiber and cell cytoskeleton interaction and position of internalized fibers, as well as the process of fiber fragmentation. The use of cancer cells might also be useful to estimate the level of cell damage and alterations that could be caused by asbestos exposure, since it is very difficult to get normal human lung epithelial cells established in vitro.

The drastic increase of multinucleated cell frequencies during the recovery times suggests a more complex mechanism of fiber-cell interaction, which would involve different end-points. So the multinucleation observed after treatment could be a consequence of the direct fiber-chromatin interaction leading to its fragmentation, or a consequence of the cytokinesis failure resulting in cells with more than one nucleus. Moreover, they could also be the result of the multipolar spindles that cannot complete the mitotic division and would originate a cell with many nuclei. The DNA histograms of multinucleated cells show an increase of nuclei from multinucleated cells with DNA content minor than 2C, data which support the suggestions above.

The aneuploidy has been associated with tumor progression, worst prognosis and high probability of tumoral relapse since the loss or gain of even one single or part of a chromosome can introduce additional mutations leading to more aggressive malignant phenotypes [11]. Also, loss or gain of chromosomes can be consequence of segregation errors and cytokinesis failure leading to high chromosome instability. Others events can cause chromosome instability, such as loss of mitotic checkpoint functions, impaired kinetochore function and centrosome amplification.

Cytokinesis failure can also generate cells with abnormal centrosome number, since the centrosome duplication occurs in G1/S [18] and in this context the cytokinesis failure can be a process involved in centrosome amplification observed in treated cells. However, some cells show a high centrosome number, like 7 centrosomes in one single cell, and in these cases the cause of the centrosome amplification can be other.

The centrosome amplification has been associated with DNA damage and mitotic checkpoints. For example, the activation of G2/M checkpoint caused by DNA damage accumulated due to a failure of DNA repair machinery can causes cell cycle arrest, and if it is long enough to allow the centrosomes (duplicated in G1) maturation, they can reduplicate in the presence of cdk2/cilcinE [25]. Together, data on the asbestos fibers potential to cause DNA damage [23] and the presence of fibers interacting with the chromatin in confocal images visualized by 3D reconstructions, suggest their association with centrosome amplification.

In cells treated with drugs that inhibit DNA synthesis, the amplification can occur during M phase by centrosome split, and after cell cycle delay caused in multinucleated cells [26]. The exposure to chrysotile fibers can cause DNA damage, and this damage can be associated with centrosome amplification too.

**Figure 5:**
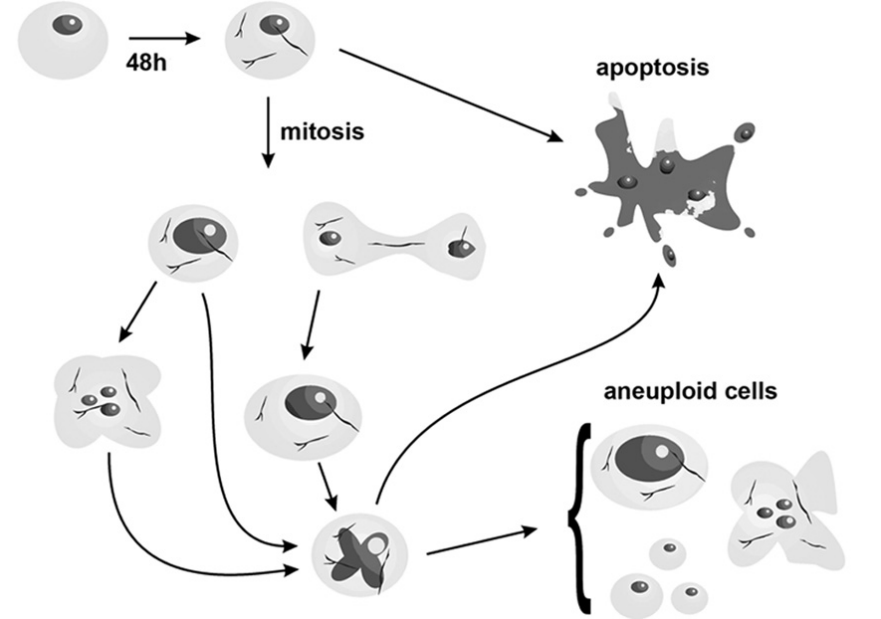
**Scheme of apoptosis, multinucleation and aneuploid HK2 cells formation after chrysotile exposure**. After 48 h chrysotile treatment the fibers are internalized and could interact with chromatin when the nuclear envelop breakdown. This interaction could lead to chromatin fragmentation or aneuploid and multinucleation cell formation due to cytokinesis failure. The cytokinesis failure form cells with centrosome amplification, that could entry mitosis with multipolar spindles and complete or not cytokinesis, leading to aneuploid cells and/or multinucleation. Apoptotic cells could be caused by checkpoint activation due several DNA or cell damage, or by centrosome amplification.

The centrosome amplification has been proposed as a mechanism of elimination of damage cells, causing the "mitotic catastrophe" [27-29]. In this context the centrosome amplification can be one of the responsible factors for apoptosis induction in chrysotile treated cells. However, the induction of apoptosis can also be a consequence of checkpoint activation due to a several DNA and cell damage (Figure 5).

## Conclusion

The present data show that chrysotile exposure for 48 h can cause centrosome amplification, apoptosis and aneuploid cell formation even when we used long periods of recovery in HK2 cells. When the nuclear envelop breakdown in early mitosis, chrysotile fibers appear to interact with the chromatin, and could lead to chromatin fragmentation and to cytokinesis failure forming an aneuploid or multinucleated cell with more than one centrosome. These cells with centrosome amplification could entry mitosis, form multipolar spindles and complete cytokinesis forming cells with abnormal DNA content due incorrect chromosomal segregation, or can incomplete cytokinesis and form one aneuploid cell with DNA content probably higher than 2C (Figure 5).

## Competing interests

The authors declare that they have no competing interests.

## Authors' contributions

BAC maintained the cell culture, performed the chrysotile treatments, prepared the samples to microscope analyses, obtained the images and drafted the manuscript. GMS conceived the study, established the confocal microscopy methodology for cell-chrysotile interaction analyses, analyzed the images and wrote the manuscript. All authors read and approved the final manuscript.

## Pre-publication history

The pre-publication history for this paper can be accessed here:



## Supplementary Material

Additional file 1 **Schematic representation of laser scanning confocal microscope configuration. A) **Fluorescein in green and propidium iodide in red; **B) **chrysotile in pink. Click here for file

Additional file 2 **Chrysotile fibers in an interphasic multinucleated HK2 cell**. After 48 h chrysotile exposure and 24 h of recovery in normal medium many fibers were found inside the HK2 cells. In interphasic cells the fibers were normally located in perinuclear region, and also in the middle of the nuclei in multinucleated cells. However, some fibers interacting with the nucleus were observed. Click here for file
