# Supplementary figures and images for: Chrysotile effects on human lung cell carcinoma in culture: 3-D reconstruction and DNA quantification by image analysis
Source: BMC Cancer. 2008 Jun 27;8:181. doi: 10.1186/1471-2407-8-181 (PMC2464777; doi:10.1186/1471-2407-8-181)

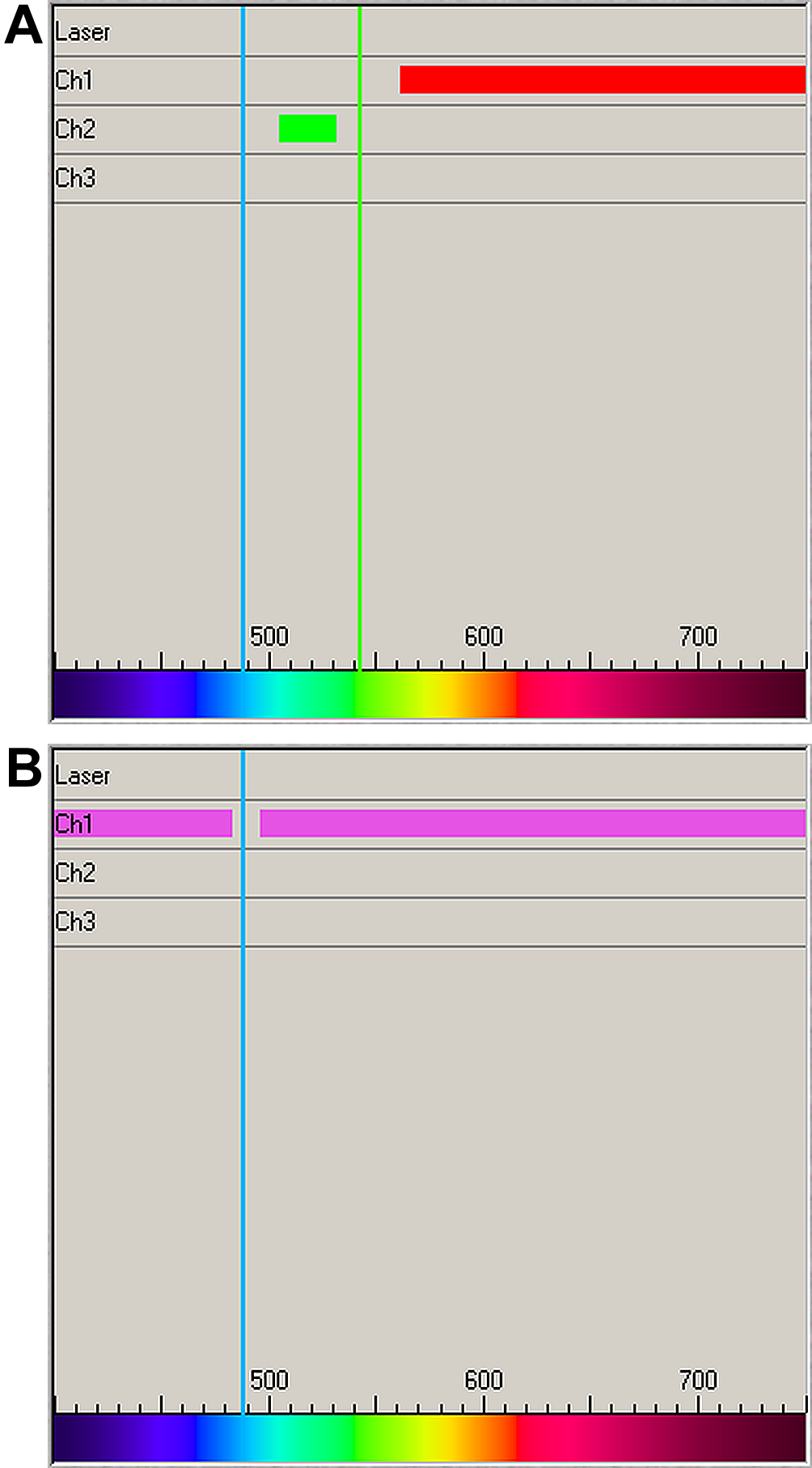

Supplement: Additional file 1 — Schematic representation of laser scanning confocal microscope configuration. A) Fluorescein in green and propidium iodide in red; B) chrysotile in pink. [file 1471-2407-8-181-S1.png]

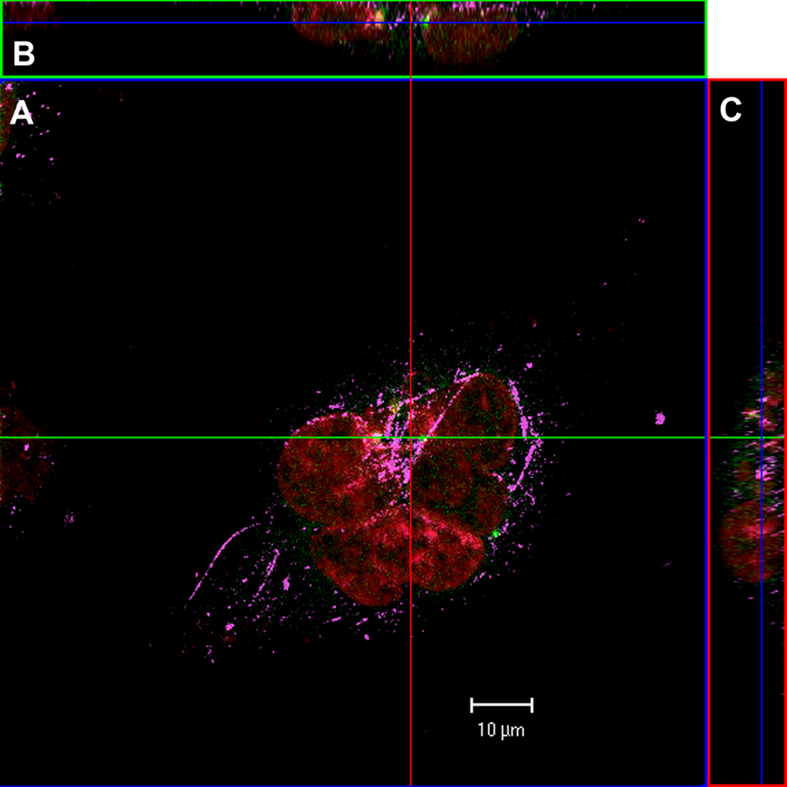

Supplement: Additional file 2 — Chrysotile fibers in an interphasic multinucleated HK2 cell. After 48 h chrysotile exposure and 24 h of recovery in normal medium many fibers were found inside the HK2 cells. In interphasic cells the fibers were normally located in perinuclear region, and also in the middle of the nuclei in multinucleated cells. However, some fibers interacting with the nucleus were observed. [file 1471-2407-8-181-S2.png]
